# 370. The comparison of adverse events in combination with double beta-lactam antimicrobials: Ampicillin plus Ceftriaxone and Ampicillin/Cloxacillin with bloodstream infections

**DOI:** 10.1093/ofid/ofad500.440

**Published:** 2023-11-27

**Authors:** Kazuhiro Ishikawa, Daiki Kobayashi, Nobuyoshi Mori

**Affiliations:** St Luke's international hospital, Chuo-ku, Tokyo, Japan; Tokyo Medical University Ibaraki Medical Center, Amicho, Ibaraki, Japan; St. Luke's International Hospital, Tokyo, Tokyo, Japan

## Abstract

**Background:**

Double beta-lactams are used to treat infective endocarditis, but the incidence rate of adverse events is unclear. We aimed to compare the incidence rates of adverse events of ampicillin(ABPC) versus ABPC plus ceftriaxone(CTRX) and ABPC/MPIPC(cloxacillin).

**Methods:**

We retrospectively included adult bacteremia patients admitted to St. Luke's International Hospital and received ABPC, ABPC plus CTRX, and ABPC/MPIPC between 2004 and 2022. We excluded patients on hemodialysis, those with missing data, those who used more than three beta-lactam antimicrobials, and beta-lactam other than ABPC, CTRX, or ABPC/MPIPC. All statistical analysis was done using IBM SPSS.

**Results:**

We included 280, 59, and 43 patients for ABPC, ABPC plus CTRX, and ABPC/MPIPC, respectively. There were significant differences in median age(70.8 [standard deviation: SD 16.6], 64.3[16.7], 56.6[17.5]), male sex(62.1%, 47.5%, 72.1%), qSOFA ≥2 (23.1%, 64.4%, 41.9%), admission in an intensive care unit(6.4%, 40.7%, 14.0%), mechanical ventilation (5.4%, 23.7%, 20.9%), use of vasopressor (10.4%, 32.2%, 23.3%), administration of vancomycin (7.5%, 27.1%, 41.9%) or clindamycin (19.6%, 23.7%, 44.2%) for more than three days, hepatic dysfunction (AST: 42.6[81.5], 120.4[493.0], 54.1[68.5], ALT: 33.1[35.9], 98.2[380.0], 46.6[67.6], anemia(Hgb: 10.8[2.1], 11.6[2.4], 11.9[2.8] and thrombocytopenia (PLT: 203.5[105.0], 154.8[115.0], 255.9[159.7]) on admission. There were significant differences in AKI (9.0%, 12.3%, 30.2%), treatment duration (13.9 [12.1] days, 9.1 [8.7] days 29.0 (19.2) days, and duration of hospitalization 34.1[31.1] days, 48.3 [50.4] days, 51.6 [32.5] days, but no difference in 30 days mortality (4.3%, 6.8%, 2.3%) and 90 days mortality (8.2%, 13.6%, 7.0%). Kaplan-Meier analysis showed no significant difference between ABPC and CTRX plus ABPC in AKI, but there was a significant difference between ABPC and ABPC/MPIPC(figure 1, 2). The hazard ratio of ABPC/MPIPC for AKI was 1.572(95% confidence interval, 1.067-2.315)(figure 3).

**Figure 1:**
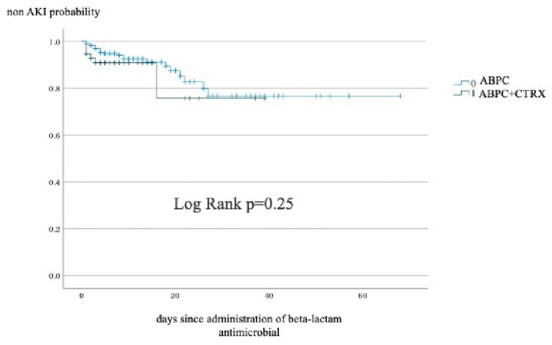
Percent of non-AKI between ABPC and ABPC plus CTRX Abbreviation: AKI, acute kidney injury; ABPC, ampicillin; CTRX, ceftriaxone

**Figure 2:**
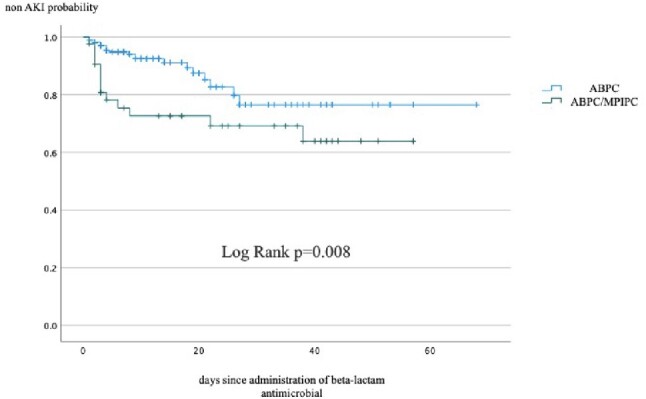
Percent of non-AKI between ABPC and ABPC/MPIPC Abbreviation: AKI, acute kidney injury; ABPC, ampicillin; MPIPC, cloxacillin

**Conclusion:**

Double beta-lactam therapy with ABPC plus CTRX may be relatively safe, but careful follow-up is needed for platelet count and hepatic function. On the other hand, ABPC/MPIPC has a significantly higher incidence of AKI compared to other antimicrobials.

Factors associated with AKI

**Figure:**
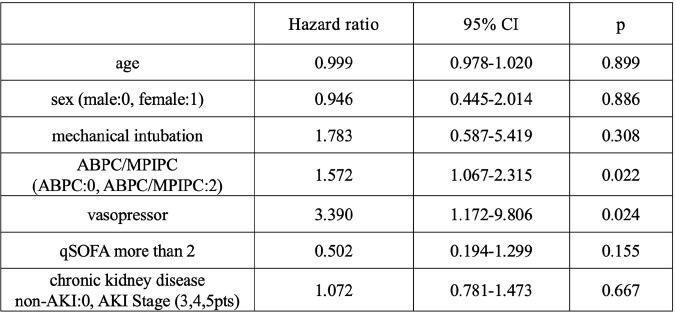

Abbreviation: AKI, acute kidney injury; ABPC, ampicillin; MPIPC, cloxacillin; CI, coincidence interval

**Disclosures:**

**All Authors**: No reported disclosures

